# A reduction in long-term spatial memory persists after discontinuation of peripubertal GnRH agonist treatment in sheep

**DOI:** 10.1016/j.psyneuen.2016.11.029

**Published:** 2017-03

**Authors:** D. Hough, M. Bellingham, I.R. Haraldsen, M. McLaughlin, J.E. Robinson, A.K. Solbakk, N.P. Evans

**Affiliations:** a Institute of Biodiversity Animal Health and Comparative Medicine, College of Medical Veterinary and Life Sciences, University of Glasgow, Glasgow G61 1QH, UK; b Department of Medical Neurobiology, Division of Clinical Neuroscience, Oslo University Hospital – Rikshospitalet, 0027 Oslo, Norway; c Division of Veterinary Bioscience and Education, School of Veterinary Medicine, College of Medical Veterinary and Life Sciences, University of Glasgow, Glasgow G61 1QH, UK; d Department of Psychology, University of Oslo, Pb 1094 Blindern, 0317 Oslo, Norway; e Department of Neurosurgery, Division of Clincial Neuroscience, Oslo University Hospital – Rikshospitalet, 0027 Oslo, Norway; f Department of Neuropsychology, Helgeland Hospital, 8651 Mosjøen, Norway

**Keywords:** Spatial memory, Hippocampus, GnRH, Puberty, Precocious puberty, Gender identity disorder

## Abstract

Chronic gonadotropin-releasing hormone agonist (GnRHa) administration is used where suppression of hypothalamic-pituitary-gonadal axis activity is beneficial, such as steroid-dependent cancers, early onset gender dysphoria, central precocious puberty and as a reversible contraceptive in veterinary medicine. GnRH receptors, however, are expressed outside the reproductive axis, e.g. brain areas such as the hippocampus which is crucial for learning and memory processes. Previous work, using an ovine model, has demonstrated that long-term spatial memory is reduced in adult rams (45 weeks of age), following peripubertal blockade of GnRH signaling (GnRHa: goserelin acetate), and this was independent of the associated loss of gonadal steroid signaling. The current study investigated whether this effect is reversed after discontinuation of GnRHa-treatment. The results demonstrate that peripubertal GnRHa-treatment suppressed reproductive function in rams, which was restored after cessation of GnRHa-treatment at 44 weeks of age, as indicated by similar testes size (relative to body weight) in both GnRHa-Recovery and Control rams at 81 weeks of age. Rams in which GnRHa-treatment was discontinued (GnRHa-Recovery) had comparable spatial maze traverse times to Controls, during spatial orientation and learning assessments at 85 and 99 weeks of age. Former GnRHa-treatment altered how quickly the rams progressed beyond a specific point in the spatial maze at 83 and 99 weeks of age, and the direction of this effect depended on gonadal steroid exposure, i.e. GnRHa-Recovery rams progressed quicker during breeding season and slower during non-breeding season, compared to Controls. The long-term spatial memory performance of GnRHa-Recovery rams remained reduced (*P* < 0.05, 1.5-fold slower) after discontinuation of GnRHa, compared to Controls. This result suggests that the time at which puberty normally occurs may represent a critical period of hippocampal plasticity. Perturbing normal hippocampal formation in this peripubertal period may also have long lasting effects on other brain areas and aspects of cognitive function.

## Highlights

• Peripubertal GnRHa impaired long-term spatial memory.
• This impairment was not reversed after discontinuing GnRHa-treatment.
• Spatial orientation and learning performance remained unaffected following GnRHa withdrawal.
• Speed of progression through these spatial tasks was altered after discontinuing GnRHa.
• GnRH irreversibly alters these cognitive functions during critical window of development.

## 1 Introduction

Gonadotropin-releasing hormone (GnRH) is a hypothalamic decapeptide that, following its release from axon terminals at the median eminence, stimulates the release of luteinizing hormone (LH) and follicle stimulating hormone (FSH) from the pituitary gland. GnRH can also reach the central nervous system (CNS), as GnRH neurones in the hypothalamus can have axons that extend into other regions of the CNS including the limbic system (Silverman et al., 1987). In addition, GnRH can cross the blood-brain barrier, from the median eminence, into the third ventricle cerebrospinal fluid, albeit with low efficiency (Caraty and Skinner, 2008). GnRH receptor expression has been demonstrated at sites within the CNS (Jennes et al., 1997, Albertson et al., 2009, Schang et al., 2011) and a range of peripheral tissues (Hapgood et al., 2005, Skinner et al., 2009). Thus, when GnRH analogs are used therapeutically in human and veterinary medicine, it is also important to consider the effects at these non-reproductive sites.

As GnRH agonists (GnRHa) result in continued receptor stimulation, as opposed to ultradian cyclic changes, theiradministration initially results in an increase in LH and FSH secretion (‘flare-effect’), followed by the down-regulation of GnRH receptor expression in the pituitary gland and suppression of reproductive axis function (Garner, 1994, Chen and Eugster, 2015). GnRHa is typically prescribed when the suppression of the reproductive axis is required, such as steroid-sensitive conditions like prostate cancer, uterine fibroids and endometriosis (Garner, 1994). In children and adolescents, GnRHa can be prescribed for treatment of central precocious puberty (CPP) (Chen and Eugster, 2015) and gender dysphoria (GD) (Hembree et al., 2009) to temporarily halt reproductive development.

Carel et al. (2009) emphasized the need for investigation of the potential psychological effects associated with peripubertal GnRHa-treatment in CPP. Similarly, the potential effects of GnRHa-treatment on cognition during this important developmental period are not well characterized. Wojniusz et al. (2016) recently demonstrated that peripubertal GnRHa increases emotional reactivity (i.e. emotional and behavioral responses to a fearful situation) in girls with CPP, whereas resting heart rate decreased and this effect was more pronounced with longer durations of GnRHa-treatment. Studies, using an ovine model, have also demonstrated that peripubertal GnRHa-treated rams display increased risk-taking behavior (Wojniusz et al., 2011), altered emotional reactivity (Evans et al., 2012) and reduced long-term spatial reference memory (Hough et al., 2016). Physiological changes within the limbic system have also been reported in this ovine model, as peripubertal GnRHa-treatment alters amygdala volume (Nuruddin et al., 2013a) and the expression of hippocampal genes that are involved in endocrine signaling and synaptic plasticity (Nuruddin et al., 2013b). With this growing body of evidence that peripubertal GnRHa-treatment may affect development of cognitive function, there is now a requirement to investigate whether these effects are reversible when GnRHa-treatment is discontinued.

In the current study, we investigated whether effects from peripubertal GnRHa-treatment, persisted in rams following the discontinuation of treatment. Specifically, we investigated whether the previously reported reduction in long-term spatial memory persists, or if effects on spatial orientation and learning emerge later in life, following the discontinuation of peripubertal GnRHa-treatment.

## 2 Materials and methods

### 2.1 Animals

All animal procedures were conducted at the University of Glasgow Cochno Farm and Research Centre (55° 55′N) under Home Office Regulations (Project License: 60/4422). The experimental animals used in this study were Scottish Mule, Texel cross males, born from same sex litters between 23 March and 12 April 2013. At birth, lambs were assigned to one of the treatment groups described below. Lambs from twin and triplet pregnancies were allocated to different groups, so that only 1 sibling was represented in each treatment group. Puberty was delayed in the GnRHa-Recovery (GnRHa-Rec, *n* = 25) lambs by subcutaneous implantation of the GnRHa, goserelin acetate (Zoladex 3.6 mg, kindly donated by Astra Zeneca, Macclesfield, UK), every four weeks from 8 to 44 weeks of age (average age of pubertal onset in male sheep is 10 weeks of age (Wood and Foster, 1998) and are expected to be sexually competent within the first year of life). Control (*n* = 30) and GnRHa-Rec rams were grazed on pasture, except during behavioral trials, when they were housed indoors with *ad libitum* access to hay or silage, with supplements as deemed necessary by standard management practices. All animals were euthanized at the end of the study period, when they were approximately 2 years of age.

### 2.2 Testes development

Approximately every four weeks, morphometric data were collected from all animals, including testes size, to monitor the effectiveness of GnRHa to suppress the reproductive axis. Scrotal length and circumference were measured with a tailor tape measure while sheep were held in a sitting-position. Testes size was calculated from the scrotal length × circumference and normalized to body weight. Testes size data at 28 (first breeding season), 44 (first anestrus), 81 (second breeding season) and 99 (second anestrus) weeks of age are presented, as these measurements were nearest to the dates of spatial maze performance assessment.

### 2.3 Assessment of spatial orientation and learning

This study used modifications of the assessment techniques and spatial mazes described previously (Hough et al., 2016), as it follows on from this study on the effects of chronic peripubertal GnRHa-treatment (with/without testosterone supplementation) on spatial orientation, learning and memory in rams from 8 to 45 weeks of age. The current study reports analyses of spatial maze performance data following the discontinuation of GnRHa-treatment at 44 weeks of age. Specifically, performance was evaluated at 83 and 95 weeks of age, and compared to that observed at 41 weeks of age (Hough et al., 2016) when the GnRH-Recovery group was still being treated with GnRHa.

#### 2.3.1 Spatial orientation and learning

**Fig. 1:**
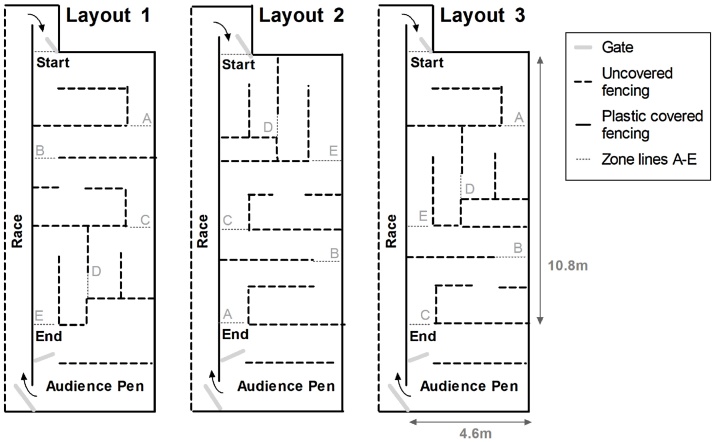
Spatial maze layouts that were used in this study, with letters indicating zones that contain the same traps across all layouts, but presented in a different order or orientation. Spatial orientation and learning was assessed in Layout 1 and 2 at 41 and 83 weeks of age, respectively. Layout 2 was used for training and the assessment of long-term spatial memory at 99 weeks of age. Layout 3 was used for the novel maze assessment at 99 weeks of age.

Sheep were individually assessed in spatial maze Layout 1 of Fig. 1 at 41 weeks of age, and in Layout 2 of Fig. 1 at 83 and 95 weeks of age. A change in maze layout was necessary, as some of the sheep were already familiar with the former layout (used in long-term spatial memory assessment at 45 weeks of age as reported by Hough et al., 2016). Each sheep was given three maze attempts within the same day (each attempt separated by ∼2 h) to traverse the maze and reunite with flock members in the audience pen. Approximately 30 sheep were assessed per day and kept in the audience pen throughout the day with *ad libitum* access to water and hay. During each maze attempt, a sheep was calmly ushered from the audience pen to the start of the maze. Sheep that failed to complete the maze within a 5 min time limit, were ushered back to the audience pen via the maze entrance so that the correct route remained unknown. On the last attempt of the day, unsuccessful sheep proceeded to the audience pen via the quickest route **Spatial orientation** was assessed as the performance of sheep in the first spatial maze attempt of the day, at each age. **Spatial learning** was assessed as the performance of sheep over three maze attempts within the same day, at each age.

#### 2.3.2 Recorded observations

Spatial performance was individually assessed by recording **traverse times** (min: s: ms), i.e. the time taken to move from the entrance to the finish line (5 min = incomplete), as well as recoding the **progress through the maze** as the time difference to move between lines A to E, judged on the placement of a front leg across the line. Emotional reactivity was not assessed in this study, as only a few vocalizations, escape attempts, urinations and defecations were observed at 83 and 95 weeks of age.

### 2.4 Assessment of long-term spatial memory

Long-term spatial memory was assessed as described previously (Hough et al., 2016).

#### 2.4.1 Training

Education and confirmation runs were performed, over two days, at 95 weeks of age, in the maze layout used in the assessment of spatial orientation and learning (Layout 2, Fig. 5). The education runs were completed when a sheep was able to traverse the maze within 1 min on two successive attempts. The retention of this ability was tested in the confirmation run, which consisted of two attempts to complete the maze within 1 min. If unsuccessful, sheep repeated the cycle of education and confirmation runs, with a maximum of 3 cycles within the same day. The total number of attempts during the education and confirmation runs was recorded for each ram, together with the quickest traverse time, to serve as a measure of the ease of training.

#### 2.4.2 Long-term spatial memory

Retention of long-term spatial memory was assessed 4 weeks after training was completed (99 weeks of age), in the same maze design. Each sheep was given one maze attempt, and traverse times (incomplete = 5 min) and progress through maze zones was recorded. The proportion of time spent in each zone was calculated for each animal as a percentage of total time spent in the maze.

#### 2.4.3 Familiarity in a novel maze design

Immediately after assessment of long-term memory, each sheep was given one attempt to traverse a new spatial maze layout (Layout 3, Fig. 1), which contained the same ‘traps’ but in a different order or orientation. Maze traverse times (incomplete = 5 min) and progress through maze zones were recorded.

### 2.5 Statistical analysis

Data were excluded from analysis where performance was judged to have been compromised because of temporary incapacity, i.e. health concerns. In addition, data were excluded from analysis where animals escaped from the maze area or jumped over internal maze walls. Exclusion of data was done by specifying a missing value for the relevant response variable(s) in that particular maze attempt (number of observations is specified in Fig. 3). All statistical analyses were performed with R software (Version 3.2.1, © 2015 The R Foundation for Statistical Computing Platform) using the RStudio interface (Version 0.99.467, © 2009–2015 RStudio Inc.). Response variables were analyzed using the generalized linear model (glm) function; ram identity was included as an explanatory variable, to account for individual variation across time or respective maze attempts.

The effect of GnRHa on body weight and normalized testes size, were analyzed using a: (1) Two-way ANOVA (Treatment × Age) in year 1 and 2, (2) Two-way ANOVA during breeding and non-breeding season. Effects of age and treatment on **spatial orientation** were assessed with data from the first attempt of the maze, across all ages (41, 83 and 95 weeks of age), using a two-way ANOVA (Treatment × Age). Effects of treatment on **spatial learning**, over three consecutive maze attempts, were assessed with two-way ANOVA (Treatment × Maze attempt) at each respective age. One-way ANOVA was used to assess the effects of treatment on the ease of **maze training** (number of training attempts at 95 weeks of age), as well as traverse times upon completion of training. Effects of treatment on **long-term spatial memory** were tested by comparison of traverse times at: (1) 99 weeks of age only (one-way ANOVA); (2) 95 (last training attempt) versus 99 (the assessment attempt) weeks of age (two-way ANOVA: Treatment × Time); and 99 (GnRHa-Recovery) versus 45 (during GnRHa-treatment) weeks of age (two-way ANOVA: Treatment × Time). The effect of maze design familiarity was examined by comparison of the traverse time of maze layouts 1 and 2 at 83 weeks of age, or layouts 2 and 3 at 95 weeks of age (two-way ANOVA: Treatment × Maze Layout). The effects of treatment and age on testes size was evaluated with a two-way ANOVA. All statistical tests were followed by a Tukey Honest Significant Difference post hoc test, to assess where significant differences existed between treatment groups. All graphs represent means and standard errors of the mean. Statistical *P*-values ≤ 0.05 were considered significant.

## 3 Results

### 3.1 Body weight and testes development

**Fig. 2:**
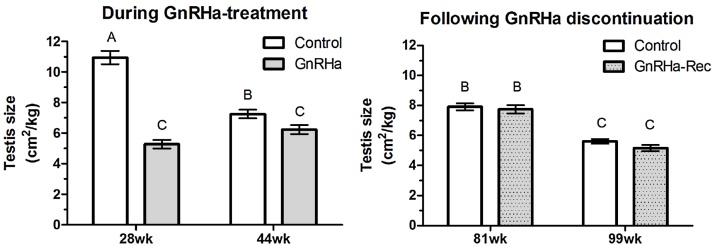
Testicular size mean ± s.e.m., as a factor of body weight, during GnRHa-treatment in the first year, and after discontinuation of GnRHa-treatment in the second year, of life relative to Control rams. Measurements were taken during the breeding seasons at 28 and 81 weeks of age. Non-breeding season measurements occurred at 44 and 99 weeks of age. Different letters on top of bars indicate significant differences in the means across both years. Control: no treatment; GnRHa: peripubertal GnRHa-treated from 8 to 44 weeks of age; GnRHa-Rec: GnRHa-treatment discontinued at 44 weeks of age.

There was no difference (*P* > 0.05) in body weight between the Control and GnRHa-treated rams at any age. Peripubertal GnRHa-treatment significantly (*P* < 0.01) suppressed normalized testes size, compared to Control rams, at both 28 and 41 weeks of age (Fig. 2). In the GnRH-Rec group, normalized testes size increased significantly (*P* < 0.001) by 25% over the 37 weeks following discontinuation of GnRHa-treatment. Normalized testes size during the breeding season in the second year of life, being 47% higher compared to the breeding season of the previous year. At 81 and 99 weeks of age, there was no difference (*P* > 0.05) in normalized testes size between Control and GnRHa-Rec groups.

### 3.2 Spatial orientation – completion of maze at the first attempt

#### 3.2.1 Traverse times

**Fig. 3:**
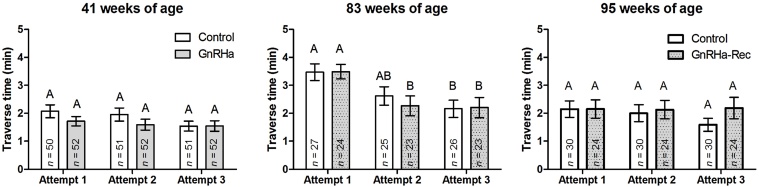
Mean ± s.e.m. traverse time of rams at 41, 83 and 95 weeks of age during spatial orientation and learning assessments, over three maze attempts within the same day. Different letters on top of bars indicate significant differences between means at that particular age. Control: no treatment; GnRHa: peripubertal GnRHa-treated from 8 to 44 weeks of age; GnRHa-Rec: GnRHa-treatment discontinued at 44 weeks of age.

The average time taken for sheep to traverse the maze (Fig. 3) was significantly (*P* < 0.001) different over the three ages tested. Compared to 41 weeks of age (Layout 1), average traverse times at 83 and 95 weeks of age were 84 and 62% slower, respectively. A different maze layout was used at 83 weeks of age (Layout 2) to that used at 41 weeks of age, and comparison of these two ages only, indicated a tendency (*P* = 0.082) for average traverse times to be longer at 83 weeks of age; an effect that was seen equally in both groups (Controls: 2.92 ± 0.29 vs. 3.47 ± 0.30 min; GnRHa-Rec: 2.96 ± 0.38 vs. 3.49 ± 0.26 min).

There were no significant effects (*P* > 0.05) of treatment or the interaction between the effects of treatment and age, on mean traverse times.

#### 3.2.2 Progress through the maze

**Fig. 4:**
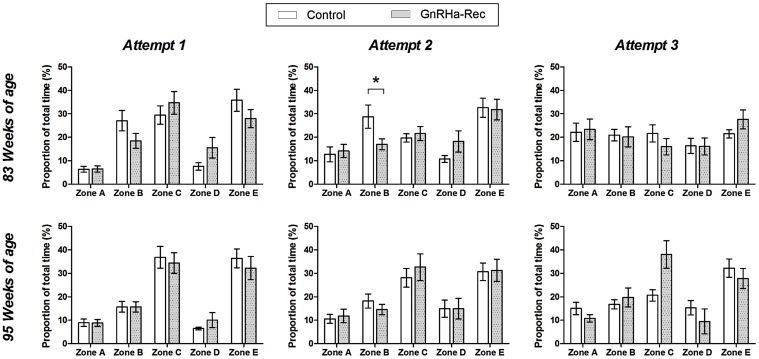
Mean ± s.e.m. proportionate time spent in each zone of the maze, expressed here as a percentage of the total time spent in the maze during spatial orientation and learning assessment at 83 and 95 weeks of age over three maze attempts. Control: no treatment; GnRHa-Rec: GnRHa-treatment discontinued at 44 weeks of age. *P < 0.05.

There were no significant effects of treatment, or interaction between the effects of age and treatment, on the proportion of time spent in any of the maze zones (Fig. 4, Attempt 1). Regardless of age (*P* > 0.05), rams spent the greatest proportion of time in zones C and E. However, rams spent significantly (*P* < 0.001) more time in zones A and B, and significantly (*P* < 0.01) less time in zone D, at 95 compared to 83 weeks of age.

### 3.3 Spatial learning *–* completion of maze with same-day repeated attempts

#### 3.3.1 Traverse times

The mean times taken to complete the maze across all three attempts within the same day, at each age, are shown in Fig. 3 and the *P*-value summary is reported in Table 1. There was no significant (*P* > 0.05) difference in spatial learning between the Control and GnRHa-Rec groups, at any age and no significant (*P* > 0.05) interaction between treatment and age. Significant improvement in traverse times over the three attempts were seen at 41 (*P* < 0.01), 83 (*P* < 0.001) and 95 (*P* = 0.050) weeks of age, but at 83 weeks of age the improvement (*P* < 0.001) was greater in the new maze layout with a 29.7% decrease in traverse times from the first to second attempt, and 10.7% from the second to third attempt.

#### 3.3.2 Progress through the maze

The mean proportion of time spent in each maze zone, for the two groups of animals, are shown in Fig. 4 with the associated *P*-value summary in Table 1. At 41 weeks of age, there was no effect of treatment on the time spent in any maze zone. At 83 weeks of age, on the first attempt, animals spent most time in zones B, C and E. On the second attempt, the proportion of time spent in each zone was more equal, with the exception of zone E, where animals spent the greatest proportion of time. By the third attempt, the time spent in each zone was approximately equal. These changes in the pattern of maze progression were reflected by a significant (*P* < 0.01) increase in the proportion of time spent in zones A (1st trap) and D (4th trap), and a significant (*P* < 0.001) decrease in the proportion of time spent in zone C (3rd trap) over the course of the three attempts. The GnRHa-Rec group spent significantly (*P* < 0.05) less time in zone B than the Control group, particularly in attempt 2. The GnRHa-Rec group also tended (*P* = 0.080) to spend less time in zone E in attempt 1, but more time in this zone in attempt 3, compared to Controls.

At 95 weeks of age, progress through the maze during the first attempt followed a similar pattern to that seen at 83 weeks of age, with the most time being spent in zones C and E. On the second attempt, there was a reduction in the proportionate time spent in these zones, together with a statistically significant increase (*P* < 0.05) in the proportion of time spent in zones A and D, but this pattern change was not as marked as seen at 83 weeks of age. The proportion of time spent in zone C tended (*P* = 0.057) to decrease with each maze attempt in the Controls, so that they spent nearly equal proportions of time across all zones by attempt 3, but the GnRHa-Rec group still spent most of their time in zones C and E, even in attempt 3.

### 3.4 Long-term spatial memory

#### 3.4.1 Traverse times

**Fig. 5:**
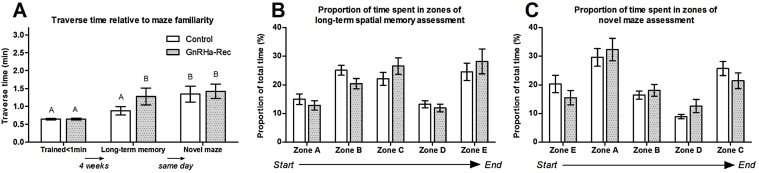
Spatial performance during long-term spatial memory and novel maze assessments at 99 weeks of age. (A) Mean ± s.e.m. traverse times after training was completed (‘Trained < 1min’), and 4 weeks later in the same familiar maze design (‘Long-term memory’) or an unfamiliar maze design (‘Novel maze’). (B) Progression through maze zones during long-term spatial memory assessment; and (C) during novel maze assessment. Different letters above bars indicate significant differences from Tukey post hoc test. Control: no treatment; GnRHa-Rec: GnRHa-treatment discontinued at 44 weeks of age.

Fig. 5A depicts the mean traverse times at the end of training (‘Trained < 1min’), and 4 weeks later when performance was assessed in the same maze (‘Long-term memory’) and in a novel maze design (‘Novel maze’). There were no effects of treatment on the number of attempts required to complete the training (Controls 6.6 ± 0.8; GnRHa-Rec 7.1 ± 0.9 attempts) or traverse times at the end of training. Compared to traverse times at the end of training, all animals took significantly (*P* < 0.001) longer to traverse the maze during long-term memory assessment, and GnRHa-treatment significantly exaggerated this effect (Treatment *P* = 0.041, Treatment × Time *P* = 0.041), as GnRH-Rec and Control animals were 2-fold and 1.3-fold slower, respectively.

When animals were tested in a novel spatial maze, the traverse times were similar (*P* = 0.564) for GnRH-Rec and Control animals. Comparison of individuals’ performances in the long-term spatial memory and novel maze assessments, indicated that all animals took significantly (Familiarity *P* < 0.001) longer to complete the novel maze, irrespective of treatment group (Treatment × Familiarity *P* = 0.146). The increase in traverse time was significantly affected by treatment (*P* = 0.008), whereby Control animals took 1.6-fold longer, and GnRH-Rec animals only took 1.1-fold longer, to complete the novel maze compared to the long-term memory assessment.

#### 3.4.2 Progress through the maze

The mean proportion of time spent in each maze zone during the long-term spatial memory and novel maze assessments are shown in Fig. 5B and C. During the long-term memory assessment, the proportion of time spent in the maze zones was not different between the GnRHa-Rec and Control groups, although there was a trend (*P* = 0.079) for the Control group to spend a proportionally greater time in zone B compared to the GnRHa-Rec group.

When animals were tested in the novel maze, progress through the maze zones was not affected by treatment, but animals spent significantly (*P* < 0.05) less time in the last three zones of the novel maze compared to the ‘familiar’ maze layout used in the long-term spatial memory assessment.

## 4 Discussion

Following the discontinuation of GnRHa-treatment at 44 weeks of age, the reproductive axis was no longer suppressed, as testes size increased to a similar size as untreated rams by 83 weeks of age and was larger at the time of the breeding versus non-breeding season. This provides evidence that endogenous GnRH and gonadal steroid signaling was restored. This delayed exposure to gonadal steroid signaling did not alter the speed at which animals completed the spatial tasks during assessments of spatial orientation (i.e. first maze attempt) and learning (progress over 3 same-day maze attempts) after peripubertal GnRHa-treatment had ceased, but affected the manner in how quickly rams moved beyond a specific point within the maze, over three same-day attempts. On assessment of long-term spatial memory at 99 weeks of age, GnRHa-Rec rams took longer to traverse a familiar spatial task, 4 weeks after training, than age-matched Controls, whereas their performance during an unfamiliar spatial task was the same as the Controls. This reduction in long-term spatial memory in GnRHa-Rec animals was also observed prior to the withdrawal of GnRHa-treatment (Hough et al., 2016) and the current study therefore reports that this reduction persisted into adulthood and was not reversed after the discontinuation of peripubertal GnRHa-treatment. A detailed discussion on these main observations follows below.

### 4.1 Spatial orientation and learning

#### 4.1.1 Prior to GnRHa withdrawal

Although peripubertal GnRHa-treatment did not have profound effects on spatial orientation (i.e. first maze attempt) and learning (i.e. change over 3 same-day maze attempts) during the first year of life (Wojniusz et al., 2011, Hough et al., 2016), peripubertal GnRHa-treatment was associated with alterations to the manner in which rams moved within the maze. This was evidenced by a decreased motivation to complete the maze and increased emotional reactivity within the maze, but did not have a significant effect on the pattern of progression through the maze zones. Supplementation of peripubertal GnRHa-treatment with exogenous gonadal steroids counteracted the effects of GnRHa on motivation and emotional reactivity.

#### 4.1.2 After GnRHa withdrawal

Given the observations in the first year of life, it is not surprising that after discontinuation of GnRHa-treatment in the second year of life, traverse times during assessments of spatial orientation and learning were unaffected in the GnRHa-Rec group. Interestingly, maze progression over three same-day attempts in the GnRHa-Rec group was quicker at 83, but slower at 95 weeks of age, compared to Controls. As breeding and non-breeding seasons are represented at 83 and 95 weeks of age, respectively, it could be indicative that, in the GnRHa-Rec rams, high gonadal steroid levels had a greater impact to improve the ability of rams to learn how to progress beyond a specific point in the spatial maze, compared to the Controls. The manifestation of these differences in maze progression patterns during the second year of life, suggests that the programming of motivational behavior and/or emotional reactivity might be dependent on exposure to gonadal steroids during a critical window of development which coincides with the peripubertal period.

### 4.2 Long-term spatial memory

#### 4.2.1 Prior to GnRHa withdrawal

As reported previously (Hough et al., 2016), during the first year of life, peripubertal GnRHa-treated rams required 1.3-fold more training attempts to learn how to complete the spatial maze than untreated rams. This effect was counteracted with testosterone supplementation, indicating that the ease of training was influenced by testosterone, rather than GnRH signaling.

Long-term spatial memory performance was also reduced in peripubertal GnRHa-treated rams, compared to Control rams (1.5-fold). As supplementation of the GnRHa-treatment with exogenous testosterone did not counteract this reduction, it indicated that long-term spatial memory is affected by the loss of GnRH, rather than testosterone, signaling.

Lastly, the assessment of rams in a spatial maze with a novel layout demonstrated that testosterone counteracted the effects of peripubertal GnRHa-treatment to reduce the spatial performance of rams. This indicated that testosterone, rather than GnRH, signaling influenced the ability of rams to solve a spatial task where familiar cues were presented in a novel sequence.

#### 4.2.2 After GnRHa withdrawal

The present study reports that, after GnRHa withdrawal the ease with which animals could be trained to complete a spatial maze in the second year of life was not different from Controls. Thus the deficit in training ability observed during peripubertal GnRHa-treatment was not permanent and a delayed exposure to gonadal steroids was sufficient to restore the speed of spatial maze training.

During the assessment of long-term spatial memory at 99 weeks of age, GnRHa-Rec rams were found to be 1.5-fold slower than Controls in traversing the spatial maze. Interestingly this difference relative to the Controls is of the same magnitude as those reported when animals were receiving GnRHa-treatment (Hough et al., 2016). The maintenance of this difference in spatial performance, following the discontinuation of peripubertal GnRHa-treatment, demonstrated that the effects of peripubertal GnRHa on long-term spatial memory were not reversed and persisted into adulthood, despite the restoration of normal GnRH and gonadal steroid signaling. This result indicates that long-term spatial memory is dependent on changes that occurred during a critical window of development that is sensitive to alterations in GnRH signaling. These changes might be related to neural plasticity and endocrine signaling as suggested by Nuruddin et al. (2013b). Spatial memory can be subdivided into long-term spatial reference memory, which categorizes spatial information according to cues that remain the same between spatial tasks (Olton and Papas, 1979) and working spatial memory, which categorizes information based on the sequence of spatial cues (Olton and Papas, 1979). As the performance of the Control and GnRHa-Rec rams were comparable at 99 weeks of age in the novel maze, which effectively tests spatial working memory, it can be concluded that the observed permanent effects of peripubertal GnRHa-treatment on long-term spatial memory is specific to spatial reference memory. It is also reasonable to argue that the increase in circulating testosterone, which should have accompanied gonadal development in the GnRHa-Rec group, must have been sufficient to eliminate any effects of the peripubertal GnRHa-treatment on spatial working memory.

### 4.3 Implications for discontinuing peripubertal GnRHa-treatment

To our knowledge, this is the first report that the effects of peripubertal GnRHa-treatment to reduce long-term spatial reference memory will persists following GnRHa withdrawal and suggests that these effects are permanent. Studies on the use of GnRHa in adult humans (Freedland et al., 2009, Green et al., 2000) have reported impaired cognition and memory loss, which were ascribed to the associated loss of estrogen and/or testosterone. Beer et al. (2006) reported that long-term memory was reduced, in terms of compromised immediate and delayed verbal memory, in men with prostate cancer and androgen deprivation (primarily GnRHa-mediated) compared to untreated healthy Controls. Another study, on elderly men with prostate cancer and GnRHa-mediated androgen deprivation (Salminen et al., 2004), reported that a decline in testosterone was associated with slower visuo-motor speed, slower reaction times relating to working memory, reduced recognition speed and delayed recall of letters, but improved object recall. These observations support the conclusion of the current ovine study that spatial working memory is reduced by the loss of testosterone, and that restoration of testosterone, either via replacement therapy (Hough et al., 2016) or gonadal development following GnRHa-withdrawal, results in normal function. However, deficits in long-term spatial reference memory did not improve with exposure to endogenous testosterone and are likely permanently altered by the changes in GnRH signaling that occurred during the peripubertal period. In so doing, it identifies the peripubertal period as a critical window of development with regard to spatial memory, in which GnRH signaling is involved. The observation that peripubertal GnRHa-treatment is associated with permanent changes in brain development raises particular concerns about the cognitive changes associated with the prolonged use of GnRHa-treatment in children and adolescents.

Limited studies have looked at the long-term effects of GnRHa-treatment on cognition in children and adolescents. One study reported that 3-year GnRHa-treatment of girls with early pubertal onset was associated with a 7% reduction in IQ (Mul et al., 2001). This effect was hypothesized to be attributed to the suppression of sex steroids and their effect on brain development which resulted in a more age-appropriate IQ (Mul et al., 2001). A recent study of girls treated with GnRHa for CPP, reported that they exhibited increased emotional reactivity and a decreased resting heart rate (Wojniusz et al., 2016). Interestingly, the changes in heart rate observed in that study were dependent upon the duration of GnRHa-treatment. Taken with the results of this study – in which it is indicated that peripubertal GnRHa-treatment affects aspects of cognitive function – it may be worth considering the duration of the GnRHa-treatment in children and adolescents, and limiting it where possible (Hayes, 2016).

The results of this study when considered with those of Hough et al., 2016 suggest that there is a critical period of brain development associated with the peripubertal period. For early onset GD, GnRHa is prescribed from childhood, throughout the adolescent period and into early adulthood, when an informed decision can be made about gender reassignment. This prolonged treatment that may encompass such critical developmental periods, however, may be justified by the increased risk of life-threatening behaviors (e.g. risk taking and suicide attempts), the effects of which could outweigh any minor cognitive and psychological impacts of GnRHa-treatment (Grossman and D’Augelli, 2007, Vance et al., 2014). For CPP, GnRHa-treatment commences from the time of diagnosis of early pubertal onset (8 or 9 years of age for girls or boys, respectively) and continues until approximately 11 years of age (Carel et al., 2009), with the main goal to increase predicted adult height by allowing more time for growth prior to the fusion of bones during puberty. However, this goal has been primarily achieved when GnRHa-treatment commenced with pubertal onset < 6 years of age (Carel et al., 2009, Hayes, 2016). The question then remains whether GnRHa exposure can be limited in children with pubertal onset between 6 to 8 or 9 years of age, or if GnRHa-treatment can be discontinued earlier than 11 years of age.

While continuous GnRHa-treatment may limit psychological problems in CPP, the decision to commence GnRHa-treatment needs further exploration (Carel et al., 2009) as proper parental and physician support might be an alternative intervention (Hayes, 2016). It has been suggested by Steinberg (2004) that a special window of susceptibility to psychosocial and psychopathological conditions exists during adolescence, which is created by the temporal gap between the onset of puberty and late adolescence. At the onset of puberty there are changes in the limbic system and increasing reward sensitivity, whereas the maturation of the prefrontal cortex is slower and age-dependent so that self-regulatory systems are only developed in late adolescence. In this regard, early-maturing children would experience a greater vulnerability towards risk-taking and novelty-seeking behavior, as well as psychopathological conditions, than children that enter puberty at an older age (Steinberg, 2004). This is evidenced by correlations between early pubertal onset and a relatively young age of first sexual intercourse, increased incidence of drug and alcohol abuse, as well as increased risk of sexual abuse (Mul and Hughes, 2008, Kim and Lee, 2012, Hayes, 2016). Against this background, the reduction in long-term spatial memory following peripubertal GnRHa, as observed in the rams in this study, might not outweigh the risks involved in withholding this treatment, but provides evidence that some cognitive functions may be irreversibly altered by peripubertal GnRHa-treatment. Further investigation on other potential cognitive and psychosocial effects are required – including whether these effects are reversible or sexually dimorphic – to assist in making informed decisions about the timing of the commencement and discontinuation of peripubertal GnRHa-treatment.

## 5 Conclusion

Spatial orientation and learning performance (i.e. traverse times) were not different from untreated rams, when assessed at 83 and 95 weeks of age, following the discontinuation of peripubertal GnRHa-treatment at 44 weeks of age. However, the effects of peripubertal GnRHa-treatment to increase emotional reactivity, persisted into the second year of life after GnRHa-treatment had been discontinued, because the manner in which rams moved through the maze (i.e. maze progress pattern) over multiple same-day maze attempts differed from the Controls. Interestingly, these aspects of how the rams progressed through a maze appeared to be dependent on the level of gonadal steroid exposure (i.e. quicker maze progression during breeding season vs. slower maze progression during non-breeding season).

The reduction in long-term spatial memory induced by peripubertal GnRHa-treatment persisted in rams into adulthood even after GnRHa-treatment was discontinued. Development of this cognitive function is, therefore, likely to occur during a critical window of development, which may reflect a time-limited period of hippocampal plasticity. Perturbations in GnRH signaling during this peripubertal period may also have long lasting effects on other brain areas and/or aspects of cognitive function.

## Figures and Tables

**Table 1:** Spatial learning. Summary of two-way ANOVA P-values to assess the effects of treatment on spatial performance across all three maze attempts within the same day at ages 41, 83 and 95 weeks. *P*-values in bold are statistically significant (*P* < 0.05) those in italics are where there is a trend (*P* < 0.1).

Response Variable	*41wks*			*83wks*			*95wks*		
	Treatment	Attempt	Treatment × Attempt	Treatment	Attempt	Treatment × Attempt	Treatment	Attempt	Treatment × Attempt
Traverse time	0.226	**0.001**	0.566	0.552	**<0.001**	0.790	0.394	*0.050*	0.440

Proportion of time									
*Zone A*	0.508	*0.076*	0.827	0.368	**<0.001**	0.734	0.450	**0.018**	0.417
*Zone B*	0.853	**0.010**	0.915	**0.021**	0.860	0.647	0.403	0.486	0.895
*Zone C*	0.273	**<0.001**	0.373	0.610	**<0.001**	0.126	0.128	*0.065*	*0.057*
*Zone D*	0.597	**<0.001**	0.783	0.441	**0.006**	0.286	0.726	**0.005**	0.812
*Zone E*	0.180	0.380	0.743	0.905	0.145	*0.080*	0.207	0.280	0.879
